# Change of Energy of the Cubic Subnanocluster of Iron Under Influence of Interstitial and Substitutional Atoms

**DOI:** 10.1186/s11671-016-1239-6

**Published:** 2016-01-12

**Authors:** Anatoliy V Nedolya, Natalya V Bondarenko

**Affiliations:** Applied Physics Department, Zaporizhzhya National University, 66 Zhukovsky str., 69600 Zaporizhzhya, Ukraine

**Keywords:** Subnanocluster, Interstitial and substitutional impurity, Octahedral interstice, Molecular mechanics method, Spontaneous growth, 61.46.-w, 61.46.Bc, 61.46.Km

## Abstract

Energy change of an iron face-centred cubic subnanocluster was evaluated using molecular mechanics method depending on the position of a carbon interstitial atom and substitutional atoms of nickel. Calculations of all possible positions of impurity atoms show that the energy change of the system are discrete and at certain positions of the atoms are close to continuous.

In terms of energy, when all impurity atoms are on the same edge of an atomic cluster, their positions are more advantageous. The presence of nickel atoms on the edge of a cubic cluster resulted in decrease of potential barrier for a carbon atom and decrease in energy in the whole cluster. A similar drift of a carbon atom from central octahedral interstitial site to the surface in the direction <011> occurred under the influence of surface factors.

Such configuration corresponds to decreasing symmetry and increasing the number of possible energy states of a subnanocluster, and it corresponds to the condition of spontaneous crystallization process in an isolated system.

Taking into account accidental positions of the nickel atom in the iron cluster, such behaviour of the carbon atom can explain the mechanism of growth of a new phase and formation of new clusters in the presence of other kind of atoms because of surface influence.

## Background

As is generally known, a new phase grows from a nucleation centre formed in local volume and has interface [1–6]. A precondition for the formation of a nucleus may be supersaturation as well as local physical changes related, for example, to the presence of soluble elements or atoms of another kind [7, 8]. Note that crystal formation is possible without large supersaturation, for example, as a result of physical changes in the local volume caused by the presence of soluble elements because of nano- and microsegregation [9, 10] owing to a concentration gradients during the accelerated crystallization. Nanosegregation processes can be viewed as changes in the nearest (first and second) environment of a certain atom, which under favourable conditions lead to further ordering of phase. These physical changes in local volume could be stimulated under influence of impurities for further crystal formation in multicomponent systems. There is a possibility of spontaneous emergence of clusters without a nucleus, their growth and self-organization of cluster groups into a crystal [11, 12]. The surface significantly affects these processes, especially for nanoscale formations [13–24].

The changes in the surroundings where the crystalline phase is created are related to the phenomenon of mass transfer [25]. The direction of mass transfer depends on the symmetry of a crystal and the type of diffusion. The rate of mass transfer depends on the properties of atoms, the property of crystalline environment, external factors, etc.

Typically, the crystallization process occurs below the equilibrium temperature of phases. But in the case of cluster formation which size corresponds to the size of a unit cell usually the molecular-kinetic approach was used than a statistical approach, according to which the size of the critical nucleus is determined by the condition of equality of opposing streams of atoms with respect to any surface of nanocrystals of this kind [26–28].

We can describe the motion of atoms in an anisotropic space by the expression with the help of a second rank tensor [29]:

1 $$ {J}_i^k=-{D}_{ij}^k{\nabla}_j{C}^k+{V}_i^{Fk}{C}^k, $$

where ${\boldsymbol{J}}_{\boldsymbol{i}}^{\boldsymbol{k}}$ is the flow of atoms of *k*th class in *i*-direction of the crystal space, ${\boldsymbol{D}}_{\boldsymbol{ij}}^{\boldsymbol{k}}$ is the factor of correlating diffusion (self-diffusion) atoms of *k*th grade, depending on the direction in the crystal, ***С***^***k***^ is the concentration of the *k*th component, and ${\boldsymbol{V}}_{\boldsymbol{i}}^{\boldsymbol{Fk}}$ is the drift velocity of the atoms of *k*th class under the external or internal force ***F*** in the *i*th direction.

The first term describes the diffusive motion of atoms and the second the drift of atoms under the external or internal influence. The drift component of atoms’ motion depends on the temperature, fields and concentration gradients, dispersion as size effect, etc.

The study of the initial stage of nanocrystalline phase formation is problematic because of transience of the process. Therefore, modelling of such processes is a real problem which attracts special attention to the research of possible stable configurations and shapes of nanocrystals [30–36].

Spontaneous crystallization process may take place only when the total free energy of the system is decreasing:

2 $$ \varDelta G=\varDelta U+p\varDelta V-T\varDelta S < 0, $$

where *Т =* const − temperature, *p =* const − pressure, *U* is the internal energy of the system, *S* is the entropy, and *V* is thevolume.

For spontaneous process in an isolated system, it is necessary that *ΔS > 0*. Taking into account at the initial moment of formation of a new phase, a nucleus volume does not change, the condition (2) simplifies to

3 $$ \varDelta U<T\varDelta S, $$

where *ΔS* at a fixed temperature can be attributed among other things also to changes of energy states of a cluster. Thus, a change in the internal energy *ΔU* should not be significant, or even be negative, which is typical for the surface layers of the new phase. For small groups of atoms, the inequality (3) may change due to statistical nature of entropy:

4 $$ \varDelta u<\varDelta Z, $$

where $\varDelta u=\frac{\varDelta U}{n}$ is the change of specific energy, $\varDelta Z=\frac{kT}{n} \ln \left(\frac{N_s}{N_c}\right)$is the thermodynamic advantage per one atom cluster when an individual atom changed its position, *n* is the number of atoms in the cluster; *N*_*c*_ and *N*_*s*_ are the number of possible states of atomic cluster changing initial central position (c) to final surface position (s) of the atom, respectively. The use of statistical entropy is justified by the fact that there was no exchange of matter at the initial time for such small isolated systems and the application of the molecular-kinetic theory is problematic.

## Methods

For the investigation, we chose a small face-centred cubic (FCC) Fe-Ni-C subnanocluster containing 15 atoms. We assumed that such a cluster forms randomly at initial time and contains one carbon atom and few nickel atoms, which substitute iron atoms. The system was considered quasi-stable and quasi-isolated, that is why it was only statics that we took into account when estimating energy using molecular mechanic method (MM+ algorithm) [37–39].

**Fig. 1:**
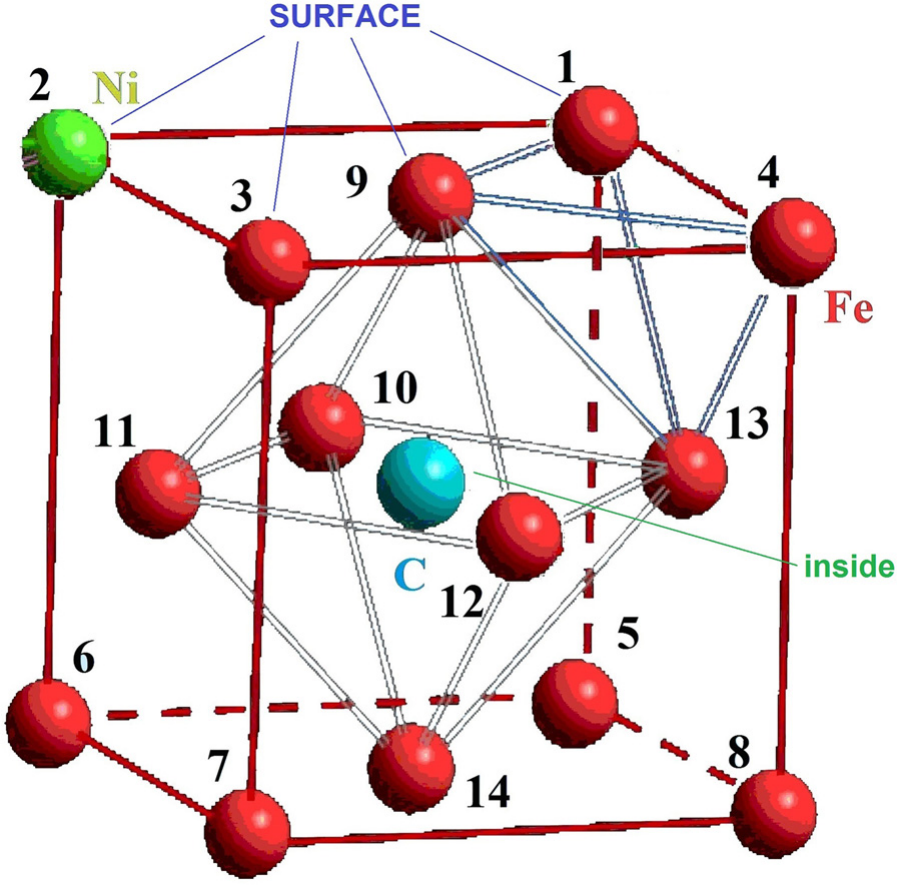
Numbering scheme of nickel atom positions in iron nanocluster

We chose an FCC cluster because all the atoms in it are located on the surface or form the surface, which simplifies interpretation of calculation results (see Fig. 1). Only carbon atom could be inside a cluster in the octahedral interstitial site as an interstitial impurity. The formation of a FCC metastable phase Fe-Ni-C is possible under non-equilibrium conditions [40, 41]. We examined every possible position of nickel atoms, which replaced the iron atoms, as an analogue of random diffused jumps of nickel atoms. We numbered their positions for convenience (Table 1). We regarded changing of carbon atom position from depth to the surface of the cluster as its drift to the surface under the influence of surface. Also, we selected the temperature of *T* = 300 K and the distances between atoms 3.6 Å (angstroms) [42, 43]. So we named this group of atoms a subnanocluster, because its size does not exceed 0.4 nm. In such a system, any changes of energy can be made only by changing positions of impurity atoms.

**Table 1:** Designation of atoms positions in FCC subnanocluster

Coordinates	[[011]]	[[001]]	[[101]]	[[111]]	[[010]]	[[000]]	[[100]]
Position	1	2	3	4	5	6	7
Coordinates	[[110]]	[[½ ½ 1]]	[[0 ½ ½]]	[[½ 0 ½]]	[[½ ½ ½]]	[[½ 1 ½]]	[[½ ½ 0]]
Position	8	9	10	11	12	13	14

We performed evaluation of energy empirically using the solution of the Newton system of equations:

5 $$ {m}_i\frac{d^2\overrightarrow{r_i}(t)}{d{t}^2}=-\frac{\partial U\left(\overrightarrow{r_i},\dots, \overrightarrow{r_n}\right)}{\partial \overrightarrow{r_i}}+\overrightarrow{F_i^{ex}}, $$

where $U\left({r}_{ij}\right)=4{\varepsilon}_{kl}{\displaystyle \sum_{i<j}\left[{\left(\frac{\sigma_{kl}}{r_{ij}}\right)}^{12}-{\left(\frac{\sigma_{kl}}{r_{ij}}\right)}^6\right]}$ is the Lennard-Jones potential (${\varepsilon}_{kl}=\sqrt{\varepsilon_{kk}{\varepsilon}_{ll}}$_,_ the bond energy, and ${\sigma}_{kl}=\frac{\sigma_{kk}+{\sigma}_{ll}}{2}$_,_ the measure of the atomic size, were calculated using Lorenz-Berthelot mixing rules of atoms of *k*th and *l*th classes) [44–47]; ${\boldsymbol{F}}_{\boldsymbol{i}}^{\boldsymbol{ex}}$ is the force that determines intermolecular interactions; *r*_*i*_ and *r*_*j*_ are the coordinates of the interacting atoms ${r}_{ij}=\left|\overrightarrow{r_i}-\overrightarrow{r_j}\right|.$

Position of a carbon atom in a central octahedral interstitial site (CIS) of a cluster was chosen as null (0) of the path length (L), conforming to central symmetry of the nanocluster, and was carried out mainly qualitative comparison of energy as the differences in specific energy *Δu* of the system, which were determined in millielectron-Volt per atom (meV/atom), considering that energy was defined up to a constant:

6 $$ \varDelta u=u(L)-u(0), $$

where *L* is the length of the carbon atom path, *u* is the specific potential energy.

## Results and Discussion

**Fig. 2:**
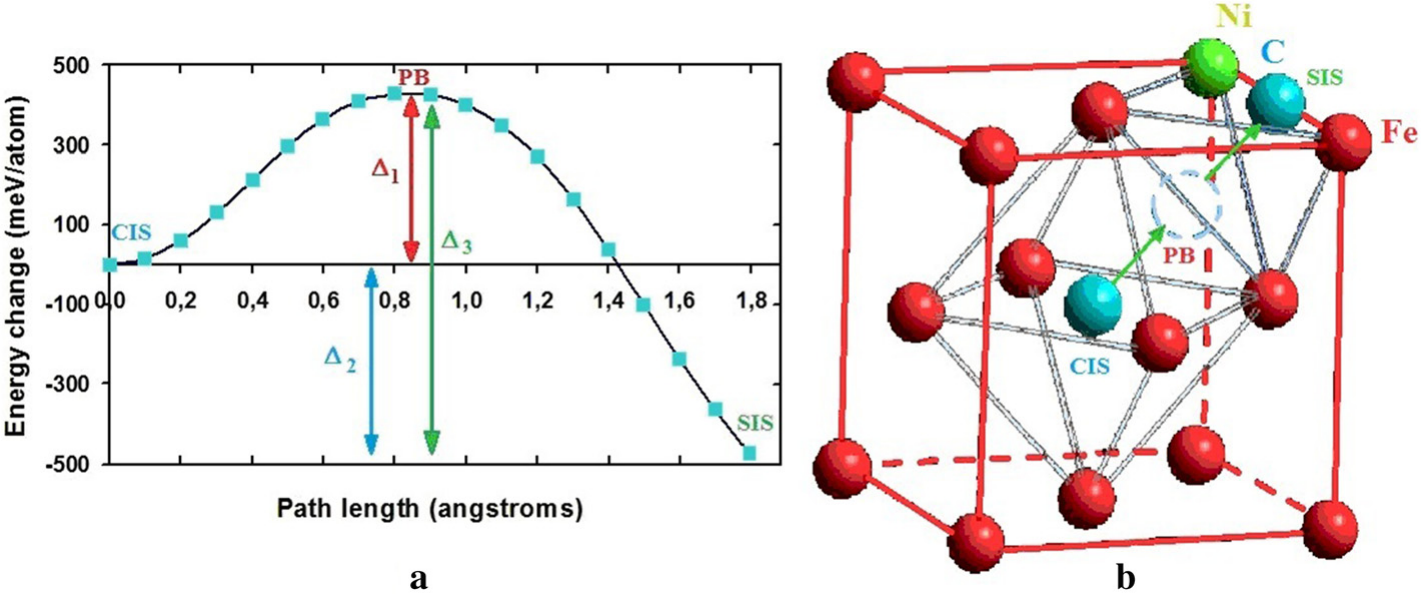
Energy evaluation criteria of FCC subnanocluster in case carbon atom changed its position. Energy evaluation criteria of FCC subnanocluster in case carbon atom changed its position from **a** central octahedral interstitial site to the **b** surface in the direction <011>

Dependence of cluster energy from position of the carbon atom (its path length) had the form of the curve with high potential barrier as indicated in Fig. 2a. The cluster energy was smaller almost twice when the carbon atom was on the surface compared to its position in the central octahedral interstice due to influence of the surface as indicated in Fig. 2b.

The estimations of energy changes of subnanocluster were based on three criteria when changing the position of the carbon atom from the central octahedral interstice in the direction of type <011> to the unfinished surface octahedral site (SIS), which is on the surface and in the middle of the cube edge:

7 $$ \begin{array}{l}{\varDelta}_1={u}_{\mathrm{PB}}\hbox{--}\ {u}_{\mathrm{CIS}},\\ {}\left|{\varDelta}_2\right|={u}_{\mathrm{SIS}}\hbox{--}\ {u}_{\mathrm{CIS}},\\ {}\left|{\varDelta}_3\right|={u}_{\mathrm{SIS}}\hbox{--}\ {u}_{\mathrm{PB}} = {\varDelta}_1+\left|{\varDelta}_2\right|,\end{array} $$

where *∆*_*1*_ is the specific barrier potential (per atom), *∆*_*2*_ is the gain in cluster energy under influence of the surface, *∆*_*3*_ is the specific changes of cluster energy between the positions of the carbon atom on the potential barrier and on the surface, *u*_*CIS*_ is the cluster’s specific energy in case when the carbon atom is in the central octahedral interstitial site, *u*_*PB*_ is the cluster’s specific energy when the carbon atom is on the maximum of potential barrier, *u*_*SIS*_ is the cluster’s specific energy in case when the carbon atom is on the surface, in the unfinished octahedral interstitial site.

### The Case of One Ni Atom

**Fig. 3:**
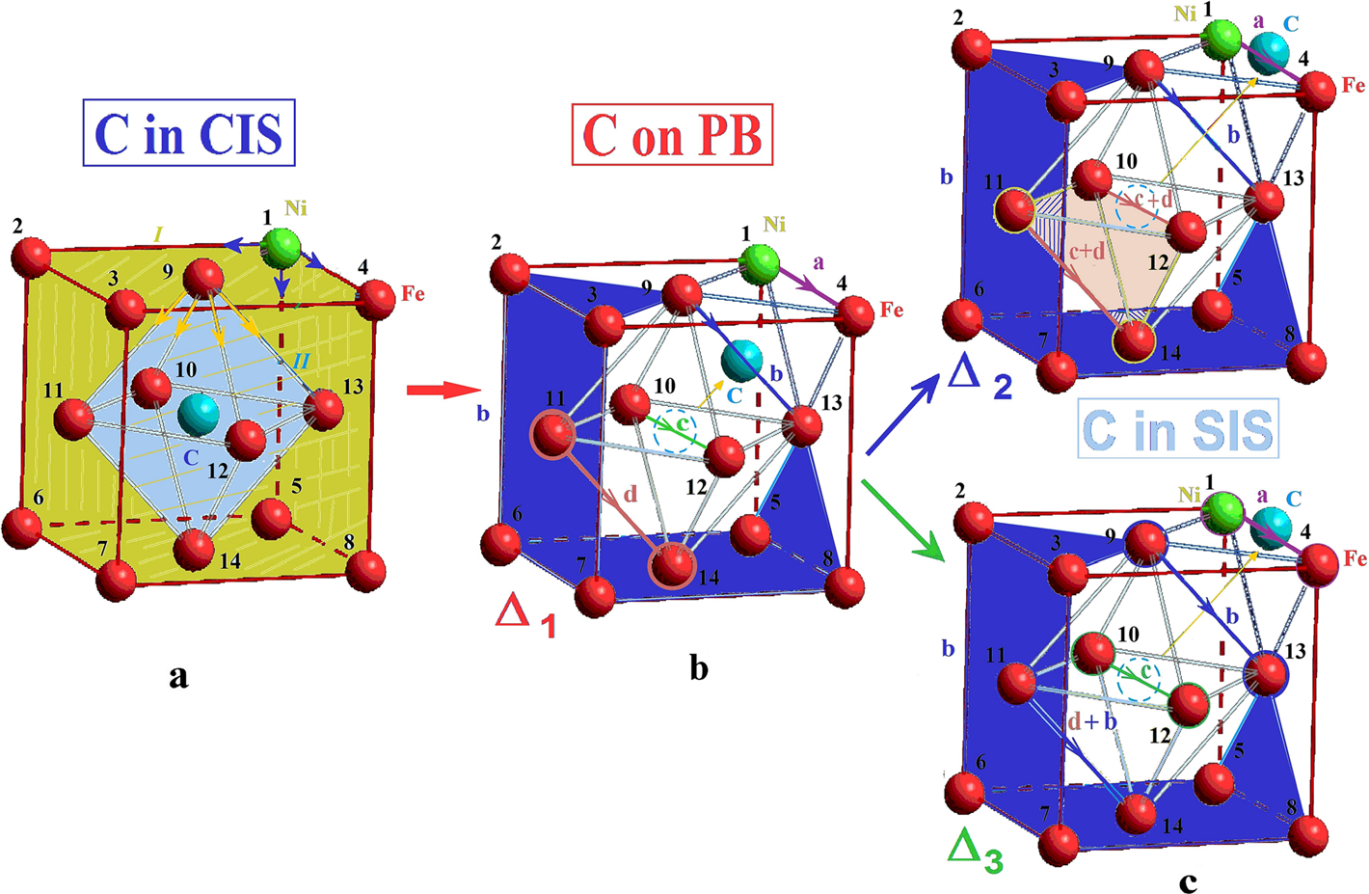
Changes of equivalent positions of a nickel atom in subnanocluster at different positions of a carbon atom. Changes of nickel atom equivalent positions in subnanocluster at different positions of carbon atom, which were assessed by Δ_1_, Δ_2_ and Δ_3_ criteria in cases carbon atom was located: **a** in the central octahedral interstitial site, **b** at the maximum of the potential barrier and **c** on the surface

There are 14 different possible positions a nickel atom may occupy substituting an iron atom. The nickel atom affects subnanocluster energy if located in the surrounding of either the first or second carbon atom, which is located in the central octahedral interstice (see Fig. 3a). It is connected with the difference of Fe and Ni atoms in size. In case when carbon atom occupies the central octahedral interstitial site, the nickel atom, which is in the first surrounding of the carbon atom (I), can take positions marked by the numbers from 1 to 8 and corresponds to plane type (002); in the second surrounding (II)—from 9 to 14 and corresponds to plane of type (111) (Table 2). The positions of the nickel atom in each surrounding of the carbon atom were energetically equivalent. The change in the nickel atom position between the first and second carbon cluster surrounding varied by 8 %.

**Table 2:** Cluster energy at different positions of carbon and nickel atoms

С in CIS	N_0_	u $\left(\frac{\mathrm{meV}}{\mathrm{atom}}\right)$	∆_1_ $\left(\frac{\mathrm{meV}}{\mathrm{atom}}\right)$	Equivalent positions	N_1_	|∆_2_| $\left(\frac{\mathrm{meV}}{\mathrm{atom}}\right)$	N_2_	|∆_3_| $\left(\frac{\mathrm{meV}}{\mathrm{atom}}\right)$	N_3_
1 ÷ 8	І	3018	427	1 = 4	a	481	a	909	a
			457	2 = 3 = 5 = 8	b	397	b	854	bd
			458	6 = 7	b	397	b	855	bd
9 ÷ 14	ІІ	2787	462	9 = 13	b	400	b	862	bd
			506	10 = 12	c	318	cd	824	c
			534	11 = 14	d	315	cd	849	bd

Cluster energy at different positions of carbon and nickel atoms where numbering is performed: *N*_*0*_—by symmetry, *N*_*1*_—by *Δ*_*1*_; *N*_*2*_—by *Δ*_*2*_; *N*_*3*_—by *Δ*_*3*_ criteria

The carbon atom can drift to the surface from the internal position of the cluster. But a carbon atom which occupies the central octahedral interstitial site of an FCC cluster has 12 directions of motion to the surface. Which direction will it choose? The answer can depend on the position of the nickel atom, which substitutes the iron atom.

**Fig. 4:**
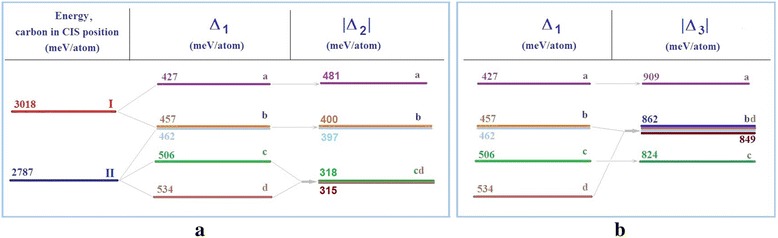
Scheme of changes of Δ_*1*_, Δ_*2*_ (**a**) and Δ_*3*_ (**b**) cluster criteria. Scheme of Δ_*1*_, Δ_*2*_ (**a**) and Δ_*3*_ (**b**) cluster criteria changes in cases positions of a nickel atom and a carbon atom changed

In case when the carbon atom changed its position from the centre to the potential barrier in the direction *<*011*>*, calculations showed that only four values of specific energy difference *∆*_*1*_ are possible depending on the positions of the nickel atom that vary by 5–10 %. The equivalent positions by the *∆*_*1*_ criteria were marked as *a*—Ni atom in position *1* or *4* (purple); *b*—Ni atom in positions *2*, *3*, *5 ÷ 9* and *13* (blue); *c*—Ni atom in position *10* or *12* (green); and *d*—Ni atom in position *11* or *14* (brown) (see Fig. 3b). In equivalent positions, the difference between specific energy changes did not exceed 1 %. This looked like splitting of differences of specific energy as indicated in Fig. 4.

A nickel atom could change the barrier potential by 20 %. The estimation of the potential barrier height shows that in the case of the Ni atom arrangement in position *1 = 4* (a), it was the lowest ~427 meV/atom, but in the case of *11 = 14*, it was the biggest ~534 meV/atom (d).

Reduction of height of the potential barrier for the carbon atom depending on the nickel atom position creates energetically favourable preconditions for the motion of the carbon atom to the surface in the direction of the nickel atom.

But due to the fact that the position of the carbon atom at the maximum of the potential barrier is unstable, the energy of more stable position of the carbon atom on the surface in unfinished octahedral interstitial site was evaluated.

In case when the carbon atom is located on the surface, the energy state of the FCC subnanocluster also depends on the nickel atom position. In this case, we evaluated specific energy changes by two criteria: in relation to the central position of the carbon atom by *∆*_*2*_ and in relation to the potential barrier by *∆*_*3*_. In both cases, the energy changes were negative, which is consistent with *∆U <* 0. Such a position of the carbon atom on the surface was more energetically advantageous than inside the cluster.

When the carbon atom is located on the surface, the quantity of Δ values reduces to three. The evaluation by *∆*_*2*_ criteria shows differences between three different values of specific energy: *a*, *b* and *cd*, because of approximation of levels *c* and *d* (see Fig. 4a). As the calculation result, we obtained *a*, *bd* and *c* levels, as a result of approximation levels *b* and *d* evaluating by *∆*_*3*_ criteria (see Fig. 4b). This looks like merging of certain levels.

**Fig. 5:**
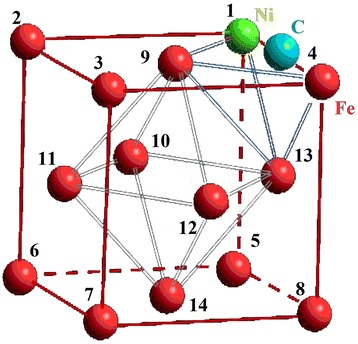
The most energetically advantageous configuration of nanocluster atoms

Our analysis shows that among all options, it is better for the carbon atom to move to the surface in direction where the nickel atom is located, because this direction is more energetically advantageous: it corresponds to the lowest energy consumption in overcoming of the potential barrier (20 %) and the largest energy gain on the surface (35 %). Thus, the configuration of the nickel atom *a* (1 = 4) produces a more desirable result and meets the condition *∆U <* 0 (see Fig. 5).

Thus, we discovered discreteness of specific energy changes depending on the nickel and carbon atoms arrangement in a FCC cluster. This is due to the changes of subnanocluster energy states as well as the change of central symmetry to axiality of the FCC cluster that meet the condition of spontaneity of the process (∆Z > 0) and depend on the individual properties of atoms (Tables 3). Such processes can be crucial during the formation of a new phase when conditions exist for its sustainable growth and joining of other atoms with the minimum loss of energy.

**Table 3:** Quantity of states of specific energy changes and their relation to statistical criteria

N_c_	N_s_	∆u $\left(\frac{\mathrm{meV}}{\mathrm{atom}}\right)$	Symbol	∆Z $\left(\frac{\mathrm{meV}}{\mathrm{atom}}\right)$	Quantity of nickel atoms in the cluster
1	3	−231	<	2.0	One nickel atom
2	6	−160	<	2.0	Two nickel atoms
		−314	<	2.0	
5	6	−147	<	0.5	Three nickel atoms
		−165	<	0.5	
		−301	<	0.5	
		−319	<	0.5	
		−466	<	0.5	

### The Case of Two Ni Atoms

In the case when a subnanocluster contains two atoms of nickel as a substitutional impurity, there are 91 variants of their placement in the cluster. However, only three positions of two nickel atoms differ by their energy: (I) both atoms are in the first surroundings of the carbon atom, (II) both atoms are in the second surroundings and (III) intermediate option, when one nickel atom is in the first and another one is in the second surroundings in case the carbon atom is in the central octahedral interstitial site of the cluster (deviation of 0.5 %). The difference between the energy values of configurations was the following: *I*–*II* 9 %; *I*–*III* 19 %; *II*–*III* 9 %, although there should be seven such positions according to cluster symmetry: *α*, *β*, *γ*, *δ*, *ε*, *ζ* and *η* (Table 4).

**Table 4:** Cluster energy at different positions of a carbon atom and a pair of nickel atoms

Equivalent positions of the Ni atoms’ pair when a С atom occupies the CIS	N_0_	u $\left(\frac{\mathrm{meV}}{\mathrm{atom}}\right)$	N_1_
1,2 = 1,4 = 2,3 = 3,4 = 1,5 = 5,8 = 4,8 = 5,6 = 6,7 = 7,8 = 2,6 = 3,7	α	2034	ІІІ
9,13 = 9,10 = 9,12 = 9,11 = 11,14 = 12,14 = 13,14 = 10,14 = 10,11 = 10,13 = 12,13 = 11,12	β	1720	І
9,14 = 11,13 = 10,12	γ	1714	І
1,13 = 4,13 = 5,13 = 8,13 = 1,9 = 4,9 = 2,9 = 3,9 = 2,11 = 3,11 = 6,11 = 7,11 = 6,14 = 7,14 = 5,14 = 8,14 = 2,10 = 1,10 = 6,10 = 5,10 = 3,12 = 4,12 = 7,12 = 8,12	δ	1880	ІІ
1,3 = 2,4 = 1,8 = 4,5 = 5,7 = 6,8 = 1,6 = 2,5 = 3,8 = 4,7	ε	2033	ІІІ
1,7 = 4,6 = 2,8 = 3,5	ζ	2033	ІІІ
6,13 = 7,13 = 5,11 = 8,11 = 1,11 = 4,11 = 2,13 = 3,13 = 6,12 = 5,12 = 2,12 = 1,12 = 7,10 = 8,10 = 3,10 = 4,10 = 3,14 = 4,14 = 2,14 = 1,14 = 6,9 = 7,9 = 5,9 = 8,9	η	1873	ІІ

Cluster energy at different positions of a carbon atom and a pair of nickel atoms, where numbering is *N*_*0*_—by symmetry, *N*_*1*_—by energy

The record *1*,*2 = 1*,*4* means that the position of the two nickel atoms of configuration *1* and *2* is the same as to the positions of *1* and *4* by the symmetry or by energy.

**Fig. 6:**
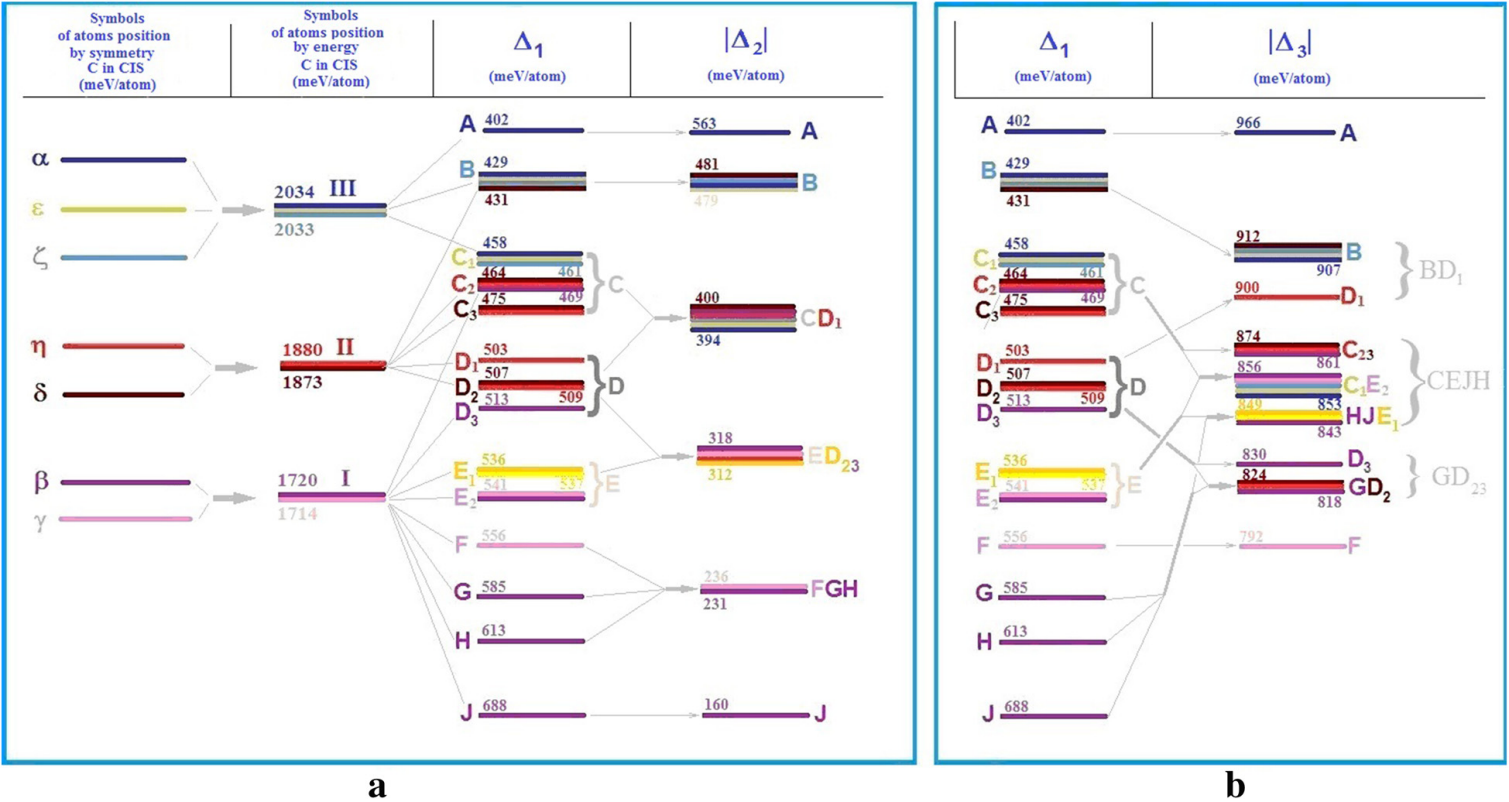
Scheme of changes by Δ_*1*_, Δ_*2*_ (**a**) and Δ_*3*_ (**b**) criteria for the case of two nickel atoms in a cluster. Scheme of changes of FCC cluster-specific energy in case Ni atoms pair and a carbon atom changed their positions compared to Δ_*1*_, Δ_*2*_ (**a**) and Δ_*3*_ (**b**) criteria

In case when the carbon was located in the potential barrier, the quantity of possible different values *Δ*_*1*_ increased to nine, which can be conditionally described as *A*, *B*, *C*, *D*, *E*, *F*, *G*, *H* and *J* levels, in the case of two nickel atoms in cluster as indicated in Fig. 6a. Small differences in the values of *Δ*_*1*_, which did not exceed 2.5 %, gave the reason to split certain levels into sublevels: *C—C*_*1*_, *C*_*2*_, *C*_*3*_; *D*—*D*_*1*_, *D*_*2*_, *D*_*3*_; and *E*—*E*_*1*_, *E*_*2*_. The difference between adjacent levels ranged from 4 to 12 %, and the maximum difference between the most remote levels was about 71 %.

The biggest barrier potential corresponded to configuration of the nickel atoms’ pair *2*,*14 = 3*,*14*. In this case, there was an increase in specific energy by 83 %. The lowest barrier potential was for configuration *1*,*4*. In this case, the energy decreased almost by 48 %. That position of the nickel atoms *1*,*4* was better for carbon atom drifting towards a smaller potential barrier.

In case when the carbon atom was on the surface, on the middle of the edge, the energy spectra of the cluster had significantly changed: the number of possible values of *Δ*_*2*_ decreased to six due to the merger of levels and sublevels into other configurations: *A*, *B*, *CD*_*1*_, *ED*_*23*_, *FGH* and *J*, depending on the position of the pair of nickel atoms. The difference between adjacent groups of values *Δ*_*2*_ was 14 ÷ 32 %. The maximum difference between the most distant values reached 72 %. The difference did not exceed 0.5 ÷ 2.0 % within of the energy level.

**Fig. 7:**
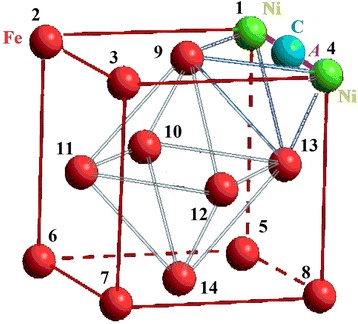
Energetically advantageous configuration of nickel atoms pair on cluster surface. Energetically advantageous configuration of nickel atoms pair at positions *1*,*4* with carbon atom on cluster surface

The highest value |*Δ*_*2*_| for the carbon atom corresponded to configuration *A* of the nickel atoms’ pair *1*,*4* (see Fig. 7), which formed a local group together with the carbon atom on the edge of the cluster. The lowest value |*Δ*_*2*_| corresponded to configuration *J* (*2,14 = 3,14*) on the opposite side of the carbon atom.

Thus, calculations show that the motion of the carbon atom towards FCC cluster surface, where the pair of nickel atoms is located, produces significant energy gain.

Evaluation by *Δ*_*3*_ criteria showed that there was a merger of levels and sublevels of the specific energy changes into five configurations with the broad value bands of *BD*_*1*_, *CEJH*, *GD*_*23*_, although the configurations of *F* and *A* were unchanged (see Fig. 6b). The differences between adjacent groups of values *Δ*_*3*_ ranged from 1.5 to 5.5 %. *Δ*_*3*_ values were in the range of 1.5 ÷ 3.5 % inside of the levels. The maximum change of *Δ*_*3*_ was 18 % between levels *A* and *F*. The lowest energy of the cluster corresponded to positions of nickel atoms (1,4) (the largest |*Δ*_*3*_|) and coincided with the same configuration by *Δ*_*2*_ criteria (Fig. 7). The iron subnanocluster was bilateral in this configuration of atoms.

Assessments are given in Table 3 with error not exceeded to 4 % in accordance with the condition (4).

It is worth nothing that the formation of energetically favourable configuration of the nickel atoms’ pair with the carbon atom on the surface of FCC nanocluster does not mean the creation of a new chemical compound that requires formation of a new surface and probably it cannot be energetically favourable. Described tendencies only mean that the presence of nickel atoms creates additional energetically favourable conditions for drift of the carbon atom exactly in the direction of the surface on the middle edge of FCC cluster where these atoms of nickel are located.

### The Case of Three Ni Atoms

During calculation analysis for three nickel atoms, we took into account 364 possible configurations of their positions in a subnanocluster.

**Fig. 8:**
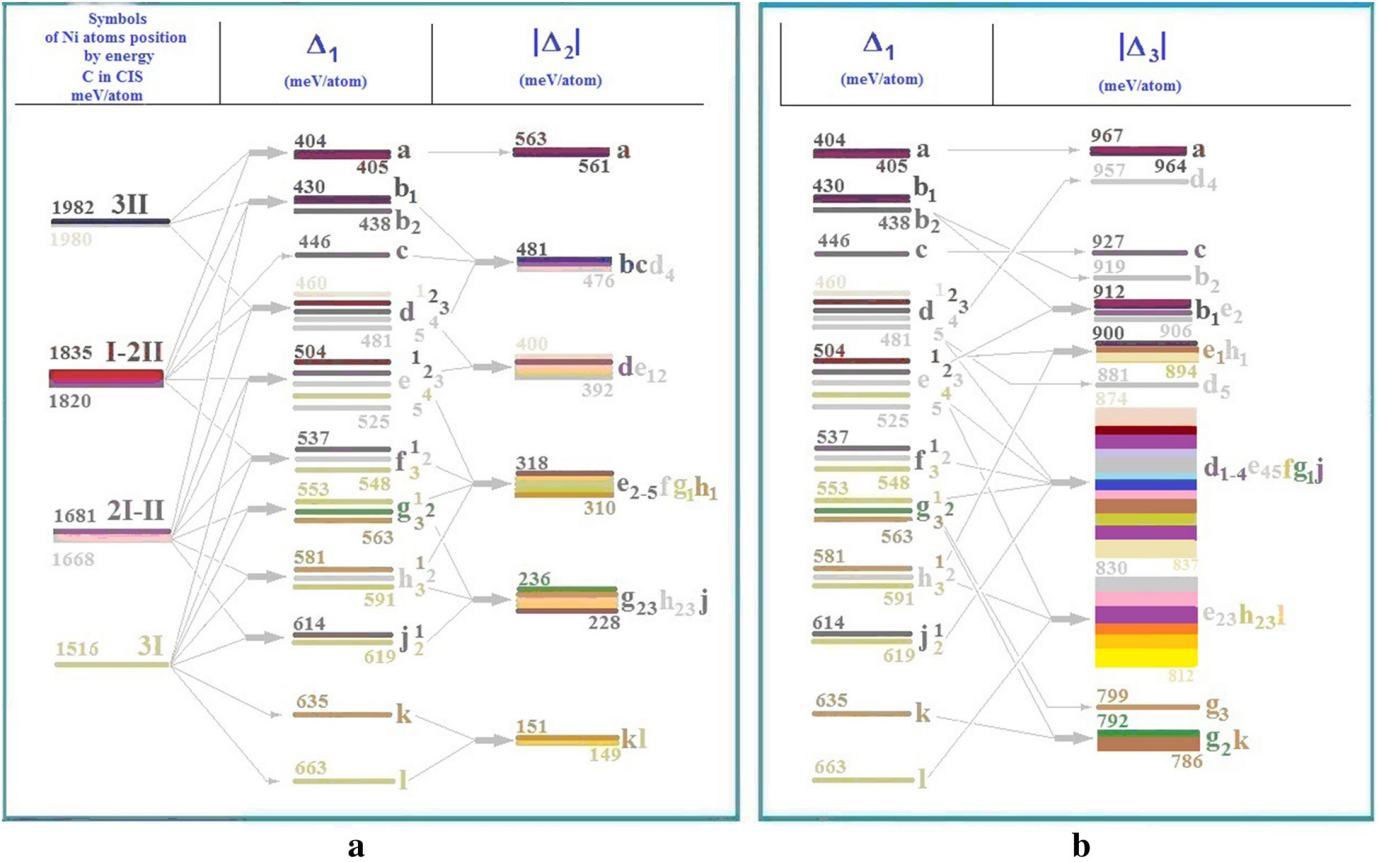
Scheme of cluster energy changes when changing positions of three Ni atoms. Scheme of FCC cluster energy changes when changing positions of three Ni atoms and a C atom compared to Δ_*1*_, Δ_*2*_ (**a**) and Δ_*3*_ (**b**) criteria

When the carbon atom was in the centre, there were only four possible values of specific energy depending on the position of the nickel atoms in the first or second surrounding of the carbon atom: *3І*, *2І*–*ІІ*, *І*–*2ІІ*, *3ІІ*. Configuration *3I* contains all variants of location of three nickel atoms in the octahedron (the first sphere of surrounding of the carbon atom). Configuration *2I–II* contains all the variants of locations of nickel atoms, where one of them is in the outer cube (second sphere) and the other two is on the octahedron (first sphere). Configuration *I*–*2II* reflects all variants of positions of nickel atoms’ pair at the apexes of the outer cube of the FCC lattice (second sphere of surrounding of the carbon atom) and the other is on the octahedron (first sphere). Configuration *3I* contains all variants of location of three atoms of nickel in the cube (the second sphere of surrounding of carbon atom) as indicated in Fig. 8.

When the position of the carbon atom changed, there was a complex picture of changes of specific energy and their splitting into levels and sublevels and their merging into broad energy bands. The maximum difference between the most distant values by *Δ*_*2*_ criteria reached 74 %, through *Δ*_*3*_ criteria is 19 %.

**Fig. 9:**
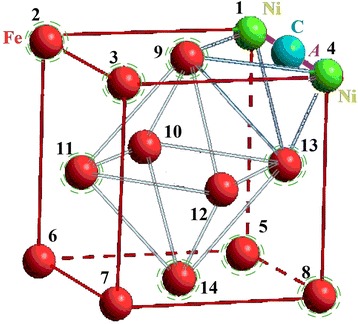
Energetically advantageous configuration of three of nickel atoms on the surface. Energetically advantageous configurations of three nickel atoms at *a* and *d*_*4*_ positions with a carbon atom on the surface

The most energetically advantageous was the position of the nickel atoms pair on the edge together with the carbon atom, and the third nickel atom could occupy one of the following positions: 2, 3, 5, 8, 9, 11, 13 and 14 (see Fig. 9). In such a cluster, the rotation-reflection symmetry axis appeared. Compliance of condition (4) for *ΔZ* was in place, although it was close to zero. It is also possible to assume that the symmetrical positions outside the cluster could be energetically advantageous in case of joining additional atoms to it.

## Conclusions

Thus, there are advantageous conditions for energy drift of interstitial impurities to the surface (carbon atom) due to asymmetric arrangement of substitutional impurities (nickel atoms) that may arise accidentally.

This configuration corresponds to the minimum energy value or even its reduction (*Δu <* 0) when the carbon atom drifts towards the nickel atoms.

These conditions with minimum energy consumption induce changes in the quantity of energy states and symmetry of the subnanocluster that increases the statistical entropy of system *ΔS >* 0.

Then, other atoms can join the surface from the lower energy side to enable the process of cluster growth. Then, the process replicates itself with the other size of cluster.

## Abbreviations

CIS: central interstitial site
FCC: face-centred cubic
PB: potential barrier
SIS: surface interstitial site
